# Metrological Evaluation of Selected Low-Cost NDIR CO_2_ Sensors for UAV-Based Air Quality Measurements

**DOI:** 10.3390/s26102988

**Published:** 2026-05-09

**Authors:** Alicja Wiora, Józef Wiora

**Affiliations:** Department of Measurements and Control Systems, Silesian University of Technology, ul. Akademicka 16, 44-100 Gliwice, Poland; jozef.wiora@polsl.pl

**Keywords:** CO_2_, UAV, measurement error, uncertainty, temperature compensation, electromagnetic interference

## Abstract

**Highlights:**

**What are the main findings?**

- Operation of UAV motors and rotors does not affect the ${\mathrm{CO}}_{2}$ readings of low-cost NDIR sensors.
- Low-cost NDIR ${\mathrm{CO}}_{2}$ sensors show large uncertainties and strong temperature dependence.

**What are the implications of the main findings?**

- Accurate ${\mathrm{CO}}_{2}$ measurements require automatic temperature compensation to avoid unrealistic readings.
- High permissible errors limit the ability of low-cost sensors to detect small ${\mathrm{CO}}_{2}$ concentration changes in air.

**Abstract:**

Air-quality measurements performed using unmanned aerial vehicles (UAVs) enable observations that are difficult or impossible to obtain with stationary monitoring systems. Although low-cost ${\mathrm{CO}}_{2}$ sensors are widely applied in such work, their accuracy is restricted by environmental influences. This study assesses the metrological performance of inexpensive NDIR ${\mathrm{CO}}_{2}$ sensors using a controlled test chamber. The TESTO probe results show strong temperature sensitivity, with ${\mathrm{CO}}_{2}$ indications varying by approximately 17 ppm per 1 °C. Measurements at −2.6 °C produced implausibly low concentrations of 275–280 ppm, despite the global baseline being about 430 ppm. Electromagnetic interference and humidity produced negligible effects on the indications. No differences appeared between measurements taken during UAV flight, after landing, or under laboratory conditions. Comparison with the manufacturer-calibrated Figaro CDM7160 sensor revealed a substantial shift in the characteristic at the lowest ${\mathrm{CO}}_{2}$ concentration level and a marked reduction in sensitivity, which shows that the sensor needs recalibration. The findings confirm that investigated low-cost ${\mathrm{CO}}_{2}$ sensors provide reasonably accurate absolute measurements only when environmental conditions are correctly compensated. However, their relatively high measurement uncertainty prevents reliable detection of small concentration changes and therefore limits their suitability for precise UAV-based air-quality studies.

## 1. Introduction

Air-quality measurements have long attracted attention from researchers and from the sociopolitical sphere. The recommendations issued by the World Health Organization (WHO) encourage many governments to take decisive actions to improve air quality [1]. A notable example is the European Union, which in the same year set targets and began actions to reduce pollutant emissions to levels safe for human health and the environment. In Poland, the National Air Protection Program, issued by the Ministry of Climate and Environment in December 2021 [2], remains in force.

Assessing progress in implementing measures to improve air quality requires continuous monitoring of selected parameters that determine air quality. The most accurate measurements are expensive and are often delayed relative to user expectations because they depend on laboratory procedures. Users typically focus on the delay between air-sample collection and access to pollutant information. The achievable measurement frequency is also important. Sensors that measure particulate-matter levels or nitrogen, carbon and sulphur oxides comply with these expectations. However, the short waiting time for measurement results often obscures the need to assess their quality. Neglecting information about measurement errors can lead to incorrect conclusions [3].

Experienced users pay attention to accuracy issues, especially when they have prior experience with measurements performed using devices moving through space. Measurement errors originate not only from the subsequent analysis of results but also from the measuring instrumentation and from the environmental conditions, under which the measurements are conducted [4]. Sensor response time, its accuracy, temperature and humidity influence, data-transmission disturbances, power supply, electromagnetic interference and sample representativeness can all significantly affect measurement results [5].

The objective of this study is to evaluate the metrological properties of low-cost Non-Dispersive Infrared (NDIR) ${\mathrm{CO}}_{2}$ sensors under varying environmental conditions, with a focus on applicability to UAV-based (unmanned aerial vehicles) air-quality measurements. This article discusses issues related to the accuracy of ${\mathrm{CO}}_{2}$ sensors and the resulting possibilities of using them to determine concentration changes aboard drones.

## 2. Background

### 2.1. Air Quality Measurements

The main source of air quality data in Poland is the Chief Inspectorate for Environmental Protection (Polish: Główny Inspektorat Ochrony Środowiska, GIOŚ). Each year, GIOŚ prepares a report assessing air quality in Poland and classifies the assessment into specific zones. The primary objective of this work is the protection of human and plant health. Pollutants monitored by GIOŚ include ${\mathrm{SO}}_{2}$, ${\mathrm{NO}}_{2}$, CO, benzene (C6H6), $O_{3}$, and particulate matter (PM) fractions ${\mathrm{PM}}_{10}$ and ${\mathrm{PM}}_{2.5}$. Concentrations of these pollutants are monitored by measurement stations distributed across the country. These stations enable online tracking of pollutant variations using dedicated sensors. Measurement values are presented hourly.

Corrections of sensor indications at the stations are performed cyclically based on reference measurements defined in Polish Standards. Laboratories also determine concentrations of pollutants contained in ${\mathrm{PM}}_{10}$, including lead (Pb), arsenic (As), cadmium (Cd), nickel (Ni), and benzo(a)pyrene. Reliable online measurements of these pollutants are not yet feasible.

Online monitoring of air pollutant concentrations began in the second half of the twentieth century. The publicly available GIOŚ database allows users to download pollution concentration data dating back to 2000, collected online and through conventional methods. Increasing public access to information and numerous environmental movements have raised awareness of hazards associated with modern civilisation. This development has led to the introduction of local smog-control regulations.

Despite existing laws and growing public awareness of the need to improve air quality, progress remains slower than expected. Many people still adopt a sceptical approach, citing disbelief in anthropogenic climate change or economic considerations [6]. For example, the heating-cost calculator on the Polish Smog Alert website indicates that coal remains one of the cheapest heating options. Moreover, non-certified household boilers enable the combustion of fuels prohibited due to high emissions of particulates and toxic gases. Inspections aimed at verifying fuel quality in stoves are infrequent and face numerous limitations. In rural communities, especially those distant from large cities with limited enforcement, such inspections are rare. Consequently, during the heating season, rural air quality is often worse than in major urban areas [7,8].

### 2.2. Measurements Using Unmanned Aerial Vehicles

A major breakthrough in air quality measurements has been the rise of UAVs, commonly known as drones. They have become widely accessible. The smallest drones, below 250 g and without cameras, are classified as objects that do not require licences or permits. However, using a drone to carry measurement systems or sensors at specific altitudes requires obtaining a UAV pilot competence certificate in the relevant category. Certificates are issued by the Civil Aviation Authority following training and a theoretical examination.

Standard drones often carry optical and thermal cameras. Some training operators now offer courses related to air quality testing, typically focused on sampling flue gases from chimneys. After modification, the sampler can be used for other measurement purposes [9]. The most common attempts at drone-based air pollution monitoring involve particulate matter, ammonia, carbon, sulphur and nitrogen oxides, volatile organic compounds (VOCs), and ozone [10]. These pollutants are primarily generated by human activity, which enables detection of pollution sources, their monitoring, or reducing their environmental impact, including eliminating the source under favourable conditions.

Measurements are typically conducted using multirotor drones with a maximum take-off mass of up to 25 kg, with payload limits determined by the drone’s construction. Payload capacity and maximum flight time are critical parameters for air quality measurements. The drone must carry sensors and the batteries that power the entire measurement system, including modules that transmit data online. The total weight determines flight time, as greater weight leads to shorter measurement duration. This limitation reduces the use of drones in long missions. Interrupting a mission to replace batteries is burdensome and costly.

One of the problems faced by people performing measurements is the correct collection of samples. The fundamental requirement is to ensure the representativeness of the measurand [11]. In the *Guide to the Expression of Uncertainty in Measurement*, non-representative sampling is identified as a key source of measurement uncertainty [12].

It is expected that air surrounding the sensors should be identical to air unaffected by the drone. Unfortunately, airflow disturbances generated by the rotors of a moving drone prevent this condition from being met. The rotors produce a strong vertical airflow that mixes air, including air directly at the sensors [13]. If the aim of the study is to create a vertical pollution profile or to determine precisely the location of a small pollution source, this problem becomes crucial. The simplest solution is to place the sampling probe at a distance from the rotors. This is most often achieved by attaching a horizontal tube to the measurement chamber, with its outlet located several tens of centimetres from the rotor’s outer edge.

It is also possible to place sampling probes above the drone. In such a case, measurements should be taken while ascending. During descent, additional vortices are generated due to the drone’s downward motion, and these do not occur during ascent [14]. In environments where a constituent can be assumed to be uniformly mixed, the impact of flow-induced disturbances on sensor indications is substantially reduced [15]. Air is a representative example of such a medium. Within the lower atmospheric layer, up to approximately 120 m above ground level, the natural spatial variability of major air components is negligibly small. Consequently, measurements of oxygen and carbon dioxide are minimally affected by localised airflow perturbations, including those induced by unmanned aerial vehicles.

### 2.3. Air Pollution Sensors

As mentioned in Section 2.1, several quantities are commonly used as indicators of air quality. Due to the increasing availability of low-cost gas sensors, individual users can monitor air quality for their own needs [16]. The primary criterion for purchasing and installing sensors in measurement systems is the relationship between product price and quality [17]. Determining the optimum point in this relationship depends on the individual needs of the user. Parameters that most often define product quality include accuracy and precision, which are determined during validation of the measurement method [17]. The prices of low-cost ${\mathrm{CO}}_{2}$ meters based on budget sensors, which offer an accuracy on the order of (10–100) ppm, and the prices of laboratory-grade analyzers, such as high-precision Picarro instruments with precision better than 1 ppm, can differ by a factor of up to 1000 or more. For low-cost gas sensors, additional attention should be paid to service life, detection limits, and selectivity.

Amperometric sensors exhibit high selectivity, but their limited lifetime and susceptibility to poisoning often disqualify them from environments heavily contaminated with volatile organic compounds [18]. Catalytic sensors are cost-effective, durable, and long-lasting, although they remain susceptible to surface contamination. However, for drone-based measurements, where energy efficiency is crucial, their heating requirement raises concerns about their usefulness [19].

NDIR sensors are primarily used for ${\mathrm{CO}}_{2}$ measurements, as ${\mathrm{CO}}_{2}$ strongly absorbs infrared radiation in the 4200–4300 nm range. These sensors offer high selectivity, long service life, good stability, and resistance to poisoning [20]. However, they are not the cheapest option and require stable optical conditions. Because of the radiation detector, these sensors are sensitive to temperature fluctuations, which makes temperature control and compensation essential. Temperature compensation is such an important yet costly issue that manufacturers of low-budget sensors often omit it and delegate this task to the user. They can also apply compensation using classical methods or machine-learning-based approaches [21]. In addition to temperature effects, reliable NDIR measurements require compensation for ambient pressure variations, as optical absorption depends on molecular number density [22]. Low-cost NDIR sensors typically do not implement explicit pressure compensation, introducing additional systematic errors under varying ambient conditions. Addressing pressure-dependent effects therefore remains a non-trivial issue, requiring more advanced calibration strategies and further investigation. Furthermore, it is also important to avoid dusty environments, which can affect optical performance.

Photoionisation detectors (PIDs) detect VOCs and some inorganic gases through photoionisation occurring inside the sensor’s measurement chamber. Because PIDs detect a wide range of compounds, they are non-selective. This feature may be advantageous when identifying the source of VOC emissions without requiring information about the specific compound present. PIDs are sensitive to contamination but are widely used in explosion-hazard atmospheric monitoring [23].

Table 1 summarises selected popular low-cost ${\mathrm{CO}}_{2}$ sensors. Their measurement ranges and stated accuracies are compared. The sensor accuracy determines the uncertainty of the measured ${\mathrm{CO}}_{2}$ concentration in air. The uncertainty $u_{B}\left(C_{\mathrm{CO}2}\right)$ was estimated for two indications. The first indication was 430 ppm, corresponding to the atmospheric ${\mathrm{CO}}_{2}$ concentration. The second indication was 3000 ppm, representing elevated concentrations.

The growing interest in low-cost air pollution sensors across many applications is confirmed by the increasing number of related publications [10]. One application involves mounting such sensors on drones to locate leaks of gases that are invisible and odourless yet dangerous to human health or life. Tracking methane plumes carried by the wind enables rapid identification of gas pipeline leak points or issues in biogas installations [24]. This procedure also requires algorithms to determine the search direction, but the final result is superior to random searching.

Similar approaches are used when mounting ${\mathrm{PM}}_{2.5}$ particulate matter sensors on drones. Locating pollution sources, especially point sources, requires algorithms that consider wind direction, wind speed, and air currents [25]. Using drones enables air quality studies both horizontally and vertically. Using multiple drones can accelerate localisation of pollution sources, although the outcome depends on cost optimisation. For this reason, such methods have not yet become widespread. The operation cost, combined with limited payload and short flight durations, restricts their practicality [26].

Mounting sensors and sampling systems on drones also enables the measurement of vertical profiles of pollutant concentrations. Mobile airborne laboratories can reach areas that are inaccessible or dangerous for humans. One example is UAV-based monitoring of volcanic ${\mathrm{CO}}_{2}$ emissions, which can support the assessment of eruption depth and intensity [27]. This form of measurement significantly enhances safety for people living in volcanic areas.

### 2.4. Research Gap

Despite growing interest in UAV-based air-quality monitoring, the metrological performance of low-cost NDIR ${\mathrm{CO}}_{2}$ sensors under real operating conditions remains insufficiently examined. Existing studies do not provide a systematic assessment of how temperature, humidity, pressure, and UAV-induced airflow jointly influence measurement accuracy. There is also a lack of comparative analyses between different low-cost ${\mathrm{CO}}_{2}$ sensors tested simultaneously in controlled laboratory conditions and during UAV flight. Current literature offers calibration or compensation methods, but their effectiveness has not been validated for sensors mounted on drones. Therefore, a clear need exists for rigorous evaluation of environmental sensitivities and practical limitations of affordable ${\mathrm{CO}}_{2}$ sensors intended for UAV applications.

## 3. ${\mathrm{CO}}_{2}$ Sensor Investigations

Thanks to advances in NDIR sensor technology, monitoring ${\mathrm{CO}}_{2}$ concentration has become relatively simple. Information about indoor ${\mathrm{CO}}_{2}$ levels is a key parameter required for effective building ventilation control [28]. However, measurement accuracy, which is critical for assessing the reliability of indicated values, is often overlooked [29].

To demonstrate the impact of measurement uncertainty under specific operating conditions, two different measurement solutions were investigated. The first solution was the TESTO 440 system (Testo 440, Testo SE & Co. KGaA, Lenzkirch, Germany), a system-level instrument equipped with a ${\mathrm{CO}}_{2}$ probe and integrated signal processing. The second solution was a Figaro NDIR ${\mathrm{CO}}_{2}$ sensor (CDM7160, Figaro Engineering Inc., Osaka, Japan) mounted on an evaluation board, representing a standalone sensor module without system-level compensation. Therefore, this study does not compare equivalent sensor types, but instead examines two distinct measurement approaches commonly used in practice.

The TESTO 440 meter for ${\mathrm{CO}}_{2}$ concentration measurement is one of many devices available on the market. Its low weight enables mounting on a drone. Airborne, with additional data logging, the device can record dynamic changes in air composition. Besides the ${\mathrm{CO}}_{2}$ sensor, the probe contains temperature, humidity, and pressure sensors, which allow the instrument to display relative humidity and dew point. The TESTO sensor manufacturer states that the permissible measurement error is ±(50 ppm+3% of reading) in the measuring range from 0 ppm to 5000 ppm. Its operating temperature range is (−5–50) °C. The meter does not provide built-in compensation for temperature, humidity, or pressure during ${\mathrm{CO}}_{2}$ measurements.

The Figaro CDM7160 ${\mathrm{CO}}_{2}$ sensor was tested as a second ${\mathrm{CO}}_{2}$ sensor type. This sensor operates within a 360–5000 ppm range. The Figaro sensor is factory-calibrated. Its specified measurement accuracy is ±(50 ppm+3% of reading). The accuracy definition is identical to that used for the TESTO probe. The operating temperature range is 0–50 °C, and the response time $t_{90}$ is 90 s. While unsuitable for outdoor measurements, its low price makes it attractive when balancing measurement capability and budget.

### 3.1. Lab Measurements

**Figure 1:**
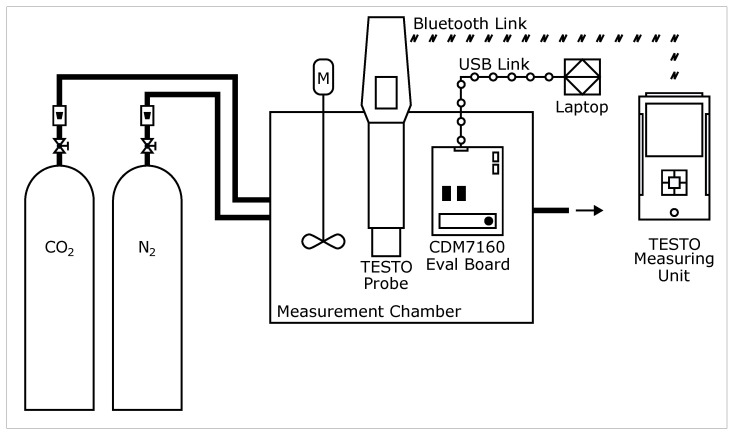
Schematic diagram of the experimental set-up used to examine ${\mathrm{CO}}_{2}$ measurement probes.

**Figure 2:**
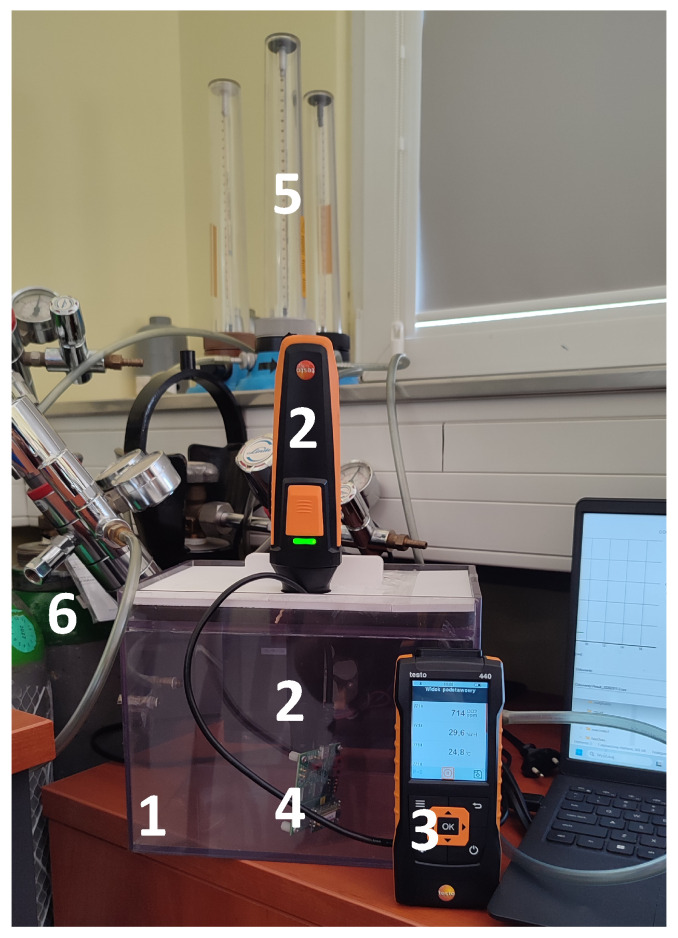
Test set-up for examining ${\mathrm{CO}}_{2}$ measurement probes—the Figaro sensor and the probe used with the TESTO 440 meter. Components of the set-up: 1—measurement chamber; 2—${\mathrm{CO}}_{2}$ measurement probe equipped with ${\mathrm{CO}}_{2}$, temperature, humidity, and pressure sensors (TESTO); 3—TESTO measuring unit (connected to the probe via Bluetooth); 4—${\mathrm{CO}}_{2}$ sensor manufactured by Figaro; 5—rotameters for controlling gas flow; 6—cylinders with nitrogen and ${\mathrm{CO}}_{2}$ calibration mixture.

The sensor tests were performed using a dedicated gas sensor test set-up, as illustrated in Figure 1. The set-up consisted of two compressed gas cylinders containing ${\mathrm{CO}}_{2}$ and $N_{2}$, supplying the measurement chamber. Gas flow rates were regulated manually using valves, while the set values were monitored using rotameters. The gases entered the chamber and were mixed electrically by an internal fan. The fan, located at the centre of the rear wall, provided continuous forced convection, ensuring homogeneous gas mixing throughout the chamber volume. The gas mixture passed over both probes and exited the chamber through small openings in the chamber wall. The signal from the TESTO probe was transmitted wirelessly to the measuring unit. The signal from the evaluation board was transmitted to a laptop via a USB cable. A photograph of the test set-up is shown in Figure 2. The internal volume of the measurement chamber was 5.2 L. All mechanical components and power supplies were located outside the chamber to minimise thermal influence on the test gas. Proper sealing prevented external air from infiltrating the chamber.

The ${\mathrm{CO}}_{2}$ measurement probe was placed inside the chamber and sealed. Calibration gas (Linde Gaz Polska Sp. z o.o., Poland), a pure mixture with a ${\mathrm{CO}}_{2}$ concentration of (3000±60) ppm, was introduced into the chamber together with ${\mathrm{CO}}_{2}$-free nitrogen. Flow control was maintained using two rotameters with an accuracy class of 2.5, corresponding to a maximum permissible error of ±2.5% of full-scale reading. The rotameters were factory-calibrated with air at 293 K and 0.1013 MPa. Manufacturer-provided temperature and pressure correction factors were applied. The combined flow of ${\mathrm{CO}}_{2}$ and $N_{2}$ was approximately $114\;{\mathrm{dm}}^{3}/h$ and remained constant throughout all measurement runs. The unit ${\mathrm{dm}}^{3}/h$ was used because the rotameters were factory-calibrated in this unit, and manufacturer-provided correction factors were applied. Under these conditions, the CO_2_ concentration stabilised after approximately 30min. This duration was adopted as the minimum stabilisation time before recording sensor indications. This value corresponded to the maximum permissible flow through the nitrogen rotameter. The gas rotameter used for ${\mathrm{CO}}_{2}$ calibration had a measurement range up to $132\;{\mathrm{dm}}^{3}/h$.

The TESTO 440 meter was powered by its internal battery. The Figaro sensor was supplied from an external power source (laptop USB), independent of the UAV power distribution system. This configuration ensured electrical isolation from motor and ESC-related noise.

NDIR sensors exhibit intrinsic response times ranging from several tens of seconds to minutes, depending on sensor design and prevailing gas diffusion conditions [20]. To determine the chamber stabilisation time, measurements were performed during transitions between minimum and maximum ${\mathrm{CO}}_{2}$ concentrations, for both increasing and decreasing steps. The response time $t_{90}$ of the chamber was 7.4 min for increasing concentration and 6.8 min for decreasing concentration. Sensor indications were recorded approximately 30 min after rotameter adjustment to ensure full stabilisation. This response time refers to stabilisation of the measurement chamber following a step change in gas concentration. It is governed by gas exchange and mixing within the chamber and does not represent the intrinsic response time of the NDIR sensors. The influence of dead volume was minimised using short, narrow tubing, an internal fan, and a sufficiently long delay after each concentration change.

The first stage of the study involved assessing the stability of the indications provided by the TESTO probe and the Figaro sensor inside a measurement chamber supplied with a constant ${\mathrm{CO}}_{2}$ concentration of 3000 ppm. The mean values of the indications differed substantially, as presented in Table 2. Type A uncertainty was estimated based on more than 100 measurements of a calibration mixture with a ${\mathrm{CO}}_{2}$ concentration of 3000 ppm, in accordance with GUM [12]:

(1) $$u_{A}^{2}\;\left(C_{{\mathrm{CO}}_{2}}\right)=\frac{1}{n(n-1)}\sum_{i=1}^{n}{\left(C_{{\mathrm{CO}}_{2},i}-{\bar{C}}_{{\mathrm{CO}}_{2}}\right)}^{2}.$$

Type B uncertainty was estimated using

(2) $$u_{B}^{2}\;\left(C_{{\mathrm{CO}}_{2}}\right)={\left(\frac{a}{\sqrt{3}}\right)}^{2}\;,$$

based on the permissible error specified by the manufacturer and assuming a rectangular probability distribution. The combined standard uncertainty of the measurement was determined using

(3) $$u_{c}\;\left(C_{{\mathrm{CO}}_{2}}\right)=\sqrt{u_{A}^{2}\;\left(C_{{\mathrm{CO}}_{2}}\right)+u_{B}^{2}\;\left(C_{{\mathrm{CO}}_{2}}\right)}.$$

The results of the uncertainty estimates for the TESTO probe and the Figaro sensor are presented in Table 2.

Combined standard uncertainty was computed from Type A estimates and Type B components, which were the dominant contributors. The low Type A uncertainty resulted from the high sensor precision, expressed by the standard deviation, relative to the larger accuracy limit defined by the permissible error.

Additionally, the influence of CO, ${\mathrm{CH}}_{4}$, and $O_{2}$ on the ${\mathrm{CO}}_{2}$ measurement was tested. As expected, the meter indications in all cases were 0 ppm.

The next step was to determine the relationship between the ${\mathrm{CO}}_{2}$ concentration in the chamber and the ${\mathrm{CO}}_{2}$ indications provided by the TESTO probe and the Figaro sensor. The ${\mathrm{CO}}_{2}$ concentration inside the chamber was calculated using the relation:

(4) $$C_{\mathrm{CO}2\mathrm{ref}}=\frac{q_{\mathrm{CO}2\mathrm{pure}}\;\cdot \;C_{\mathrm{CO}2\mathrm{pure}}+q_{N2}\;\cdot \;C_{\mathrm{CO}2\mathrm{inN}2}}{q_{\mathrm{CO}2\mathrm{pure}}+q_{N2}}=\frac{q_{\mathrm{CO}2\mathrm{pure}}}{q_{\mathrm{CO}2\mathrm{pure}}+q_{N2}}\;\cdot \;C_{\mathrm{CO}2\mathrm{pure}},$$

where: $C_{\mathrm{CO}2\mathrm{ref}}$—reference ${\mathrm{CO}}_{2}$ concentration in the measurement chamber (ppm), $q_{\mathrm{CO}2\mathrm{pure}}$—flow rate of certified 3000 ppm ${\mathrm{CO}}_{2}$ mixture (${\mathrm{dm}}^{3}$/h), $C_{\mathrm{CO}2\mathrm{pure}}$—${\mathrm{CO}}_{2}$ concentration in the certified mixture = 3000 ppm, $q_{N2}$—nitrogen flow (${\mathrm{dm}}^{3}$/h), $C_{\mathrm{CO}2\mathrm{inN}2}$—${\mathrm{CO}}_{2}$ concentration in nitrogen = 0 ppm. For the flow measurements *q*, a correction factor accounting for ambient pressure and temperature was applied. Table 3 lists the volumetric flow rates for both gases.

The combined standard uncertainty $u_{c}\;\left(C_{\mathrm{CO}2\mathrm{ref}}\right)$ was calculated according to

(5) $$\begin{array}{c} u_{c}^{2}\left(C_{\mathrm{CO}2\mathrm{ref}}\right)={\left[\frac{q_{\mathrm{CO}2\mathrm{pure}}}{q_{\mathrm{CO}2\mathrm{pure}}+q_{N2}}\;\cdot \;u_{c}\left(C_{\mathrm{CO}2\mathrm{pure}}\right)\right]}^{2}\\ +{\left[C_{\mathrm{CO}2\mathrm{pure}}\;\cdot \;\frac{q_{N2}}{{\left(q_{\mathrm{CO}2\mathrm{pure}}+q_{N2}\right)}^{2}}\;\cdot \;u_{c}\left(q_{\mathrm{CO}2\mathrm{pure}}\right)\right]}^{2}\\ +{\left[C_{\mathrm{CO}2\mathrm{pure}}\;\cdot \;\frac{q_{\mathrm{CO}2\mathrm{pure}}}{{\left(q_{\mathrm{CO}2\mathrm{pure}}+q_{N2}\right)}^{2}}\;\cdot \;u_{c}\left(q_{N2}\right)\right]}^{2} \end{array}$$

incorporating uncertainties of gas concentration and flow. Uncertainty contributions associated with the rotameter accuracy class were treated as systematic components and included in the Type B uncertainty of the reference CO_2_ concentration.

**Figure 3:**
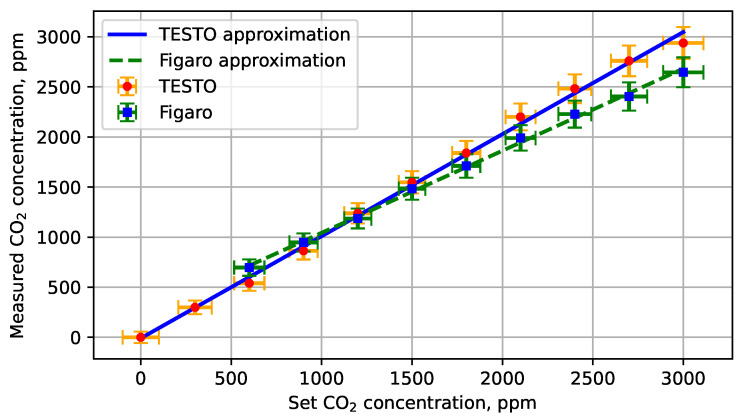
Characteristics of the TESTO probe and the Figaro sensor. The data used to construct the characteristics are provided in Table 3. Linear trend coefficients for the TESTO probe: a=1.019, b=−9.909. Linear trend coefficients for the Figaro sensor: a=0.820, b=223.1.

Figure 3 shows the relationship between the set ${\mathrm{CO}}_{2}$ concentration and the measured value. Verification was conducted at 28 °C and 996.7 hPa. The ambient laboratory temperature remained at (23 ± 1) °C and was monitored continuously throughout all measurements. Relative humidity remained within the operational range of the humidity sensor. Comparison of the Figaro sensor characteristics with the reference reveals systematic component, including zero error and sensitivity error. The Figaro sensor measurement range is 360–5000 ppm. Indications below 360 ppm ${\mathrm{CO}}_{2}$ are inaccurate and were therefore excluded from the analysis. The sensor is intended for installation in indoor air quality monitoring systems. Correction of systematic components should be implemented at the measurement system design stage to minimise systematic bias.

The observed intercept offset represents a systematic component rather than a random component. Potential contributors include sensor ageing, baseline drift after prolonged inactivity, and characteristics of the manufacturer-supplied evaluation board and firmware. Recent software- and hardware-based compensation methods have been reported [20,21]; however, their implementation was beyond the scope of this study. The purpose of this work was to identify the magnitude and metrological consequences of bias, rather than to optimise sensor calibration.

The literature states that ${\mathrm{CO}}_{2}$ indications are significantly affected by temperature. The TESTO sensor does not include built-in automatic temperature compensation. This omission results in measurements taken near 0 °C showing unrealistically low outdoor ${\mathrm{CO}}_{2}$ concentrations. Globally, outdoor ${\mathrm{CO}}_{2}$ concentration remains about 430 ppm, regardless of season or location (source: https://www.co2.earth/daily-co2, accessed on 15 March 2026).

Measurements taken at −2.6 °C, 1002.9 hPa, and 47.4% relative humidity showed indications between 275 ppm and 280 ppm. Such values are not plausible. The manufacturer specifies that the probe can operate between −5 °C and +50 °C.

Given this discrepancy, additional tests were conducted at 7 °C, 23 °C, and 38 °C. For temperature-dependent measurements, the measurement chamber and sensors were placed inside a climatic chamber. Gas cylinders and rotameters were located outside at a stable laboratory temperature. Consequently, temperature variations did not affect gas flow control.

**Figure 4:**
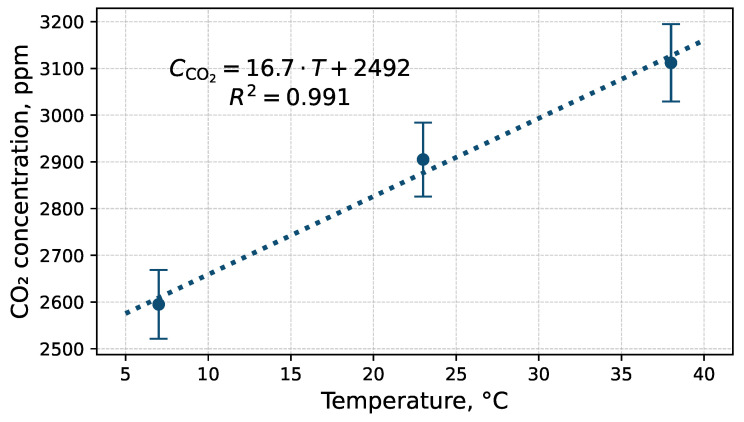
Influence of temperature on the indications of the TESTO probe. The ${\mathrm{CO}}_{2}$ concentration in the measurement chamber was 3000 ppm ± 60 ppm. $u_{c}\left(C_{\mathrm{CO}2\mathrm{measT}}\right)=\sqrt{u_{A}^{2}\left(C_{\mathrm{CO}2\mathrm{measT}}\right)+u_{B}^{2}\left(C_{\mathrm{CO}2\mathrm{measT}}\right)}$.

Tests of the TESTO sensor showed that a 1 °C change in temperature alters the indicated ${\mathrm{CO}}_{2}$ concentration by approximately 17 ppm, as shown in Figure 4. During UAV flight, the air temperature near the sensor may vary with altitude or due to thermal gradients generated by the underlying surface. These temperature variations can influence the sensor’s internal optical and electronic components, altering the infrared absorption characteristics and affecting the indicated ${\mathrm{CO}}_{2}$ concentration. Consequently, temperature compensation is essential to ensure measurement validity, even though the resulting indications may still fall within the sensor’s specified permissible error margin.

Temperature sensitivity was determined under quasi-static conditions after thermal stabilisation. Dynamic temperature step-response tests were not conducted and are therefore considered a limitation of this study. The reported temperature coefficient should not be extrapolated to rapidly changing thermal conditions encountered during dynamic flight.

**Figure 5:**
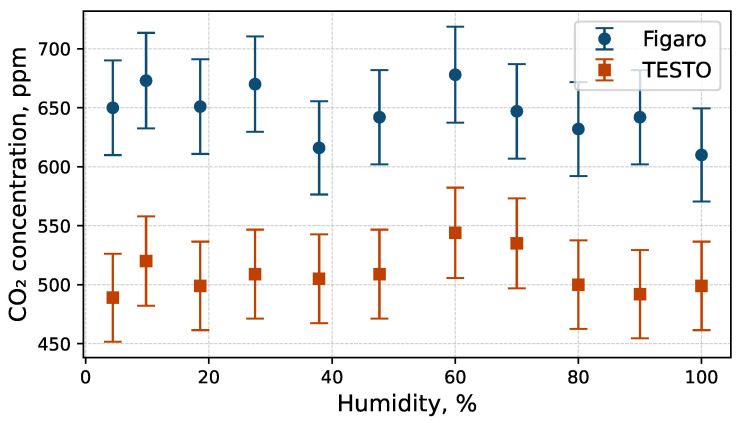
Influence of humidity on ${\mathrm{CO}}_{2}$ indications of the TESTO probe and the Figaro sensor. No clear correlation was observed.

Figure 5 shows the dependence of indications from both sensors on relative humidity. Measurements were taken at 24 °C and an ambient pressure of 1000 hPa. The influence of humidity on ${\mathrm{CO}}_{2}$ indications was investigated using a humidity calibrator (General Eastern; Model C–1 RH Generator) equipped with a gas dryer and a water reservoir. Relative humidity was monitored using a calibrated Delta Ohm HD 2101.1 thermohygrometer as a reference instrument. All humidity tests were conducted at a constant temperature to ensure well-defined relative humidity conditions.

Neither sensor demonstrates high accuracy; therefore, their sensitivity to humidity is limited and indications remain within acceptable error thresholds. However, a nearly constant offset of approximately 140 ppm is observed between the two sensors. This offset is consistent with the calibration results shown in Figure 3.

Condensation on optical components may occur when the sensor environment crosses the dew point, increasing light scattering and causing measurement errors. Such conditions were not encountered during the present experiments and were therefore not investigated. The reported results apply to operating conditions without condensation and should not be extrapolated to fog, cloud, or supersaturated environments.

### 3.2. Field Measurements

**Figure 6:**
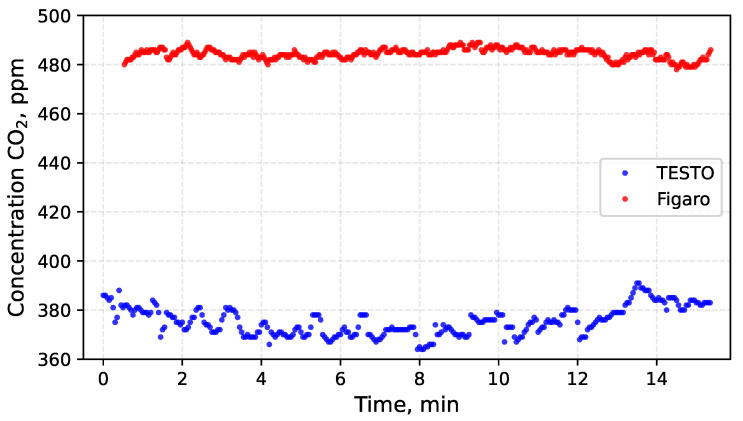
Measurement of atmospheric ${\mathrm{CO}}_{2}$ concentration using the TESTO probe and the Figaro sensor during UAV flight was carried out to compare their operational performance.

**Figure 7:**
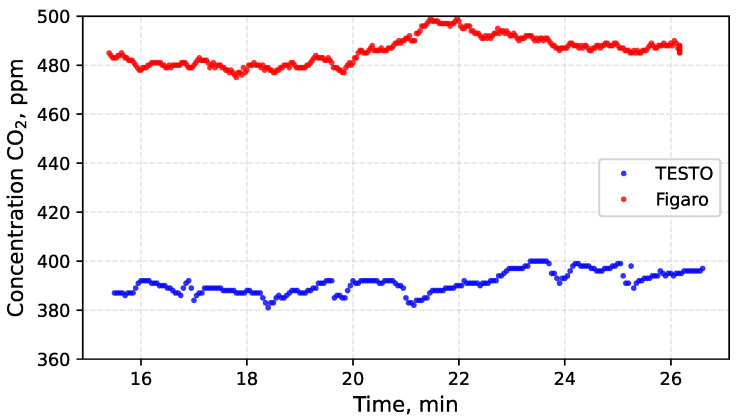
Measurement of atmospheric ${\mathrm{CO}}_{2}$ concentration using the TESTO probe and the Figaro sensor after landing with the motors switched off was performed to assess their behaviour.

The TESTO measurement probe and the Figaro sensor were mounted on the UAV platform. Electrical interference from motors and ESCs was minimised by using electrically isolated power supplies for both measurement devices. A flight experiment was conducted to evaluate the impact of disturbances generated by UAV operation, including rotor motion and motor activity. To minimise airflow variability around the sensors, measurements were performed while hovering approximately 1 m above the ground at a wind-sheltered location. The results of the measurements obtained during flight are presented in Figure 6 and Figure 7. Subsequently, ${\mathrm{CO}}_{2}$ measurements were also performed on the ground with the UAV motors switched off.

The mean ${\mathrm{CO}}_{2}$ concentrations recorded during motor operation and in the stationary state differ between the TESTO probe and the Figaro sensor. However, in all cases the values remain within the permissible error range specified by the manufacturers. The observed increase in ${\mathrm{CO}}_{2}$ concentration after several minutes results from gradual stabilisation of airflow around the UAV, previously disturbed by rotor-induced turbulence. This interpretation is supported by the accompanying changes in ambient temperature. At the beginning of the flight, the temperature was approximately 20 °C and subsequently decreased to about 18.5 °C. Toward the end of the measurements, it returned to approximately 20 °C. During stationary ground measurements, the temperature increased to 21 °C. As demonstrated earlier, temperature fluctuations exert a measurable influence on ${\mathrm{CO}}_{2}$ indications.

The Figaro sensor includes built-in temperature compensation, whereas the TESTO probe does not possess this feature. Consequently, the mean ${\mathrm{CO}}_{2}$ concentration indicated by the TESTO probe increased by 16 ppm (from 375 ppm to 391 ppm), as shown in Table 4. The corresponding change observed in the Figaro sensor amounted to only 2 ppm (from 484 ppm to 486 ppm).

To assess the impact of disturbances induced by UAV operation, the standard deviation of the ${\mathrm{CO}}_{2}$ measurements was adopted as the indicator of variability. A comparison of the standard deviation values recorded during flight and during stationary operation reveals only minor differences. For the TESTO probe, the standard deviation during flight reached 6 ppm ${\mathrm{CO}}_{2}$, decreasing to 4 ppm once the motors were switched off. In the case of the Figaro sensor, the changes were similarly small: the standard deviation amounted to 3 ppm during flight and 6 ppm during stationary operation. For both sensors, the observed variations in standard deviation σ appear to result primarily from the stabilisation of airflow and temperature after landing rather than from the direct influence of UAV operation itself.

## 4. Conclusions

The growing availability of unmanned aerial vehicles capable of carrying sensors, actuators, and cameras enables measurements in locations previously inaccessible to humans. However, enthusiasm for technological innovation may obscure the need to verify the validity and practical value of specific measurements or resulting actions. Lack of awareness of measurement errors may lead to incorrect conclusions. Many everyday users of measuring instruments assume that displayed values are unquestionably accurate.

As demonstrated in this study, ${\mathrm{CO}}_{2}$ concentration indications are affected by substantial measurement errors. The Figaro sensor exhibited high precision but significant systematic bias, which requires recalibration before use in quantitative air quality measurements. The observed uncertainties make it impossible to detect subtle ${\mathrm{CO}}_{2}$ changes reliably using tested low-cost sensors and meters. In applications such as building-ventilation control, these errors remain acceptable.

For drone-mounted sensors, only large and rapid ${\mathrm{CO}}_{2}$ changes can be detected reliably, such as those occurring during fires. Combining ${\mathrm{CO}}_{2}$ sensors with ${\mathrm{PM}}_{2.5}$ sensors could accelerate the detection of early fire-ignition hotspots that are invisible to thermal cameras. Sensor packages with real-time data transmission offer a cost-effective alternative when a camera is already installed on the drone.

Temperature effects were characterised under stabilised, quasi-static conditions. Humidity did not exert a significant influence on the indications of ${\mathrm{CO}}_{2}$ concentration, and the fluctuations in ${\mathrm{CO}}_{2}$ indications remained within the permissible error range of the sensors. A similar behaviour can be observed in the measurements taken during UAV flight and after landing with the motors switched off. The standard deviation values recorded during flight and during stationary operation differ by only 2–3 ppm, and they remain at the level observed under laboratory conditions. This indicates that the investigated low-cost ${\mathrm{CO}}_{2}$ sensors are not observably affected by disturbances from UAV motors or propellers under hovering conditions.

A detailed analysis of the metrological properties of ${\mathrm{CO}}_{2}$ sensors enables the development of correction methods that compensate for errors caused by varying conditions. When real-time compensation is not possible, corrections may be applied during post-processing. This approach increases the usefulness of lower-accuracy sensors in applications that require higher resolution or greater precision.

## Figures and Tables

**Table 1:** Selected low-cost ${\mathrm{CO}}_{2}$ sensor types with key parameters and calculated Type B standard uncertainty components for two concentration levels.

${\mathrm{CO}}_{2}$ Sensor Type	Measurement Range	Accuracy	$u_{B}\;\left(C_{\mathrm{CO}2}\right)$ @430 ppm	$u_{B}\;\left(C_{\mathrm{CO}2}\right)$ @3000 ppm
Sensirion SCD41 ^1^	400–5000 ppm	±(40 ppm+5% of reading)	36 ppm	110 ppm
Senseair S8 ^2^	400–10,000 ppm	±(40 ppm+3% of reading)	31 ppm	75 ppm
Figaro CDM7162-C00 ^3^	360–5000 ppm	±(50 ppm+3% of reading)	36 ppm	81 ppm
Sensirion SCD30 ^1^	400–10,000 ppm	±(30 ppm+3% of reading)	25 ppm	69 ppm
Amphenol Telaire T6713 ^4^	0–5000 ppm	±(30 ppm+3% of reading)	25 ppm	69 ppm
Senseair K30 ^2^	0–5000 ppm	±(30 ppm+3% of reading)	25 ppm	69 ppm
Cubic CM1106-C ^5^	0–5000 ppm	±(50 ppm+5% of reading)	41 ppm	115 ppm
GSS CozIR-LP2 ^6^	0–5000 ppm	±(30 ppm+3% of reading)	25 ppm	69 ppm

^1^ Sensirion AG, Stäfa, Switzerland; ^2^ Senseair AB, Delsbo, Sweden; ^3^ Figaro Engineering Inc., Osaka, Japan; ^4^ Amphenol Telaire, Goleta, CA, USA; ^5^ Cubic Sensor and Instrument Co., Ltd., Wuhan, China; ^6^ Gas Sensing Solutions Ltd., Cumbernauld, UK.

**Table 2:** Components of the combined uncertainty for ${\mathrm{CO}}_{2}$ concentration measurements reported for the TESTO probe and the Figaro sensor. Precision is expressed by the standard deviation (σ), and accuracy is expressed by the maximum permissible error (MPE). The standard uncertainties $u_{A}\left(C_{{\mathrm{CO}}_{2}}\right)$, $u_{B}\left(C_{{\mathrm{CO}}_{2}}\right)$, and the combined standard uncertainty $u_{c}\left(C_{{\mathrm{CO}}_{2}}\right)$ are reported. The measurements were performed in a chamber at a temperature of 30 °C. The ambient pressure was 996.7 hPa. The ${\mathrm{CO}}_{2}$ concentration in the chamber was 3000 ppm ± 60 ppm. The measurement duration was 15 min. The sampling interval was 2 s for the Figaro sensor and 3 s for the TESTO probe.

	${\bar{C}}_{{\mathrm{CO}}_{2}\mathrm{meas}}$ ppm	σ ppm	MPE ppm	$u_{A}\left(C_{{\mathrm{CO}}_{2}}\right)$ ppm	$u_{B}\left(C_{{\mathrm{CO}}_{2}}\right)$ ppm	$u_{c}\left(C_{{\mathrm{CO}}_{2}}\right)$ ppm
TESTO	2971	5	139	0.6	80	80
Figaro	2497	9	125	0.3	72	72

**Table 3:** Values of the gas flow rates indicated by the rotameters, together with the set and measured ${\mathrm{CO}}_{2}$ concentrations obtained from the Testo (T) and Figaro (F) sensors, including the associated measurement uncertainties for both the reference and the measured ${\mathrm{CO}}_{2}$ concentration.

$q_{N2}$ dm^3^/h	$q_{\mathrm{CO}2\mathrm{pure}}$ dm^3^/h	$C_{\mathrm{CO}2\mathrm{ref}}$ ppm	$u_{c}\left(C_{\mathrm{CO}2\mathrm{ref}}\right)$ ppm	$C_{\mathrm{CO}2\mathrm{measT}}$ ppm	$u_{c}\left(C_{\mathrm{CO}2\mathrm{measT}}\right)$ ppm	$C_{\mathrm{CO}2\mathrm{measF}}$ ppm	$u_{c}\left(C_{\mathrm{CO}2\mathrm{measF}}\right)$ ppm
114	0	0	50	0	29	– *	–
101	13	300	46	299	34	–	–
92	22	600	42	540	38	696	41
80	34	900	39	864	44	947	45
68	46	1200	37	1238	50	1186	49
58	58	1500	37	1547	56	1483	55
46	68	1800	39	1840	61	1711	59
34	80	2100	42	2200	67	1990	63
23	91	2400	45	2482	72	2228	67
12	102	2700	50	2760	77	2404	71
0	114	3000	55	2938	80	2645	75

* The symbol (–) indicates data exceeding the measurement range of the sensor.

**Table 4:** Comparison of the ${\mathrm{CO}}_{2}$ concentration measurement results (mean ${\mathrm{CO}}_{2}$ concentration) obtained using the TESTO probe and the Figaro sensor on the UAV during flight and after landing with the engines switched off, including the standard deviation of the ${\mathrm{CO}}_{2}$ concentration measurements.

	${\bar{C}}_{{\mathrm{CO}}_{2}\mathrm{measT}}$ ppm	$\sigma_{T}$ ppm	${\bar{C}}_{{\mathrm{CO}}_{2}\mathrm{measF}}$ ppm	$\sigma_{F}$ ppm
During flight	375	6	484	2
After landing	391	4	486	6

## Data Availability

The data presented in this study are available on request from the corresponding author.
